# Sero-prevalence of HIV infection among tuberculosis patients in a rural tuberculosis referral clinic in northern Nigeria

**Published:** 2010-06-21

**Authors:** Grace Pennap, Stephen Makpa, Sam Ogbu

**Affiliations:** 1 Microbiology Unit, Nasarawa State University, Keffi, PMB 1022 Keffi, Nasarawa State, Nigeria; 2 TB/Leprosy Referral Center, Evangelical Reformed Church of Christ, Alushi, Nasarawa State, Nigeria

**Keywords:** HIV, Tuberculosis, co-infection, Nigeria

## Abstract

Co-infection with Human Immunodeficiency virus (HIV) and Mycobacterium tuberculosis the causative agent of Tuberculosis (TB), has been referred to as the “cursed duet” as a result of the attendant morbidity and mortality due to their synergistic actions. This study was carried out to determine the prevalence of HIV infection among Tuberculosis (TB) confirmed patients on admission at a TB referral centre. The association of HIV prevalence with gender and age as risk factors was also determined. Blood samples were collected by venipuncture from 257 TB patients and their HIV status determined. Viral antibody detection was carried out using ELISA kits which detected both HIV-1 and HIV-2 and confirmed by Western blot. Of the 257 patients screened, 44.20% (106) were HIV positive. The prevalence of co-infection was higher among the female (44.82%) than the male (38.30%) patients and highest among those aged 21-40 years old (45.30%). Co-infection was found to be statistically highly associated with gender and age (p<0.05). A very high prevalence of HIV infection was reported in this study among patients that were on admission on the grounds that they had only TB. It is therefore important to screen for HIV among all TB patients.

## Letter to the editors

An estimated 1/3 of the world’s population is infected with Mycobacterium tuberculosis, with the highest prevalence of the disease found in sub-Saharan Africa and Asia. More than half of these live in countries ravaged by HIV/ AIDS [1]. However, Nigeria has been noted as among the leading countries burdened by the scourge and even ranks 4^th^ among the 22 countries that account for 80% of the world’s TB cases [2].

Similarly, the emergence of Human Immunodeficiency Virus (HIV) has paved way for the resurgence of Mycobacterium tuberculosis infection. HIV is the most powerful risk factor for the progression of M. tuberculosis infection to TB disease. Being infected with both HIV and M. tuberculosis is the world’s leading cause of death due to infectious agents [3].

The two are intricately linked to malnutrition, unemployment, poverty, drug abuse and alcoholism and have also been referred to as the “Cursed Duet” [1]. HIV is known to increase the risk of reactivation in people with latent tuberculosis and also increases the risk of subsequent episodes of TB from exogenous reinfection. HIV patients are highly vulnerable to TB because of their weakened immune systems and the latter is now their number one killer. Surveillance of HIV among TB patients has been recognised as important as the HIV epidemic continues to fuel TB epidemics. Reports show that in Sub-Saharan Africa, HIV sero-prevalence rates among TB patients range from 2467% [1].

In the view of the aforementioned, this study became imperative in order to provide baseline data in Nasarawa State to alert the TB control programs of the potential HIV problems with a view to the development of joint strategies. More so that anti tuberculosis treatment has been shown to be complicated by frequent drug interactions with highly active antiretroviral therapy (HAART) and adverse drug reactions are more common among HIV-infected patients [1].

The study was carried out between March 2007 and August 2008 among 257 patients admitted and receiving treatment for TB.

Blood samples were collected and tested for HIV-I and HIV-2 using a chromatographic qualitative ELISA test kit according to the manufacturer’s instructions. All ELISA positive samples were further confirmed by Western blot.

Of the 257 TB patients tested for HIV infection, 106 were positive giving a prevalence of 41.2% infection in this group. This is the highest rate that has been reported for such studies in Nigeria. Reports of similar studies in Nigeria reported 12.0% in Ile-Ife [4], 10.0% in Kano [5], 10.5% and 14.9% among children and adults respectively in Sagamu [6] and 28.12% in Ibadan [7]. In relation to gender, 44.83% (52/116) of the female patients tested positive while 38.30% (54/141) of the male patients tested positive for HIV infection. This difference was found to be statistically significant (p<0.05). This is probably related to the higher incidence of HIV infection usually found in females than males which could have predisposed more of them to TB as the former is known to activate dormant TB. Women are also known to have a higher susceptibility to HIV infection and are usually exposed to sexual activities earlier than men mainly due to economic circumstances. Furthermore, most African women are so subordinated to their husbands that they have little or no say in issues related to sexual relationships [6]. It is therefore possible for one male to have been the source of infection to several females. This could have also been the reason for the preponderance of HIV infection among the female patients. Interestingly, this study was carried out in an environment where polygamy thrives a lot. Although in their study of sex differences in the clinical presentation of urban Nigerian patients with pulmonary tuberculosis, Lawson and colleagues [8] noted that women were more likely to be co-infected with HIV than males while Odaibo et al.[9] did not observe any significant difference in the rate of co-infection in relation to gender.

Based on the findings of this study there is a high HIV prevalence in TB patients. This is of great concern especially as it might affect both patient management and public health prospective. It therefore underscores the need for routine HIV serology on all TB patients so as to reduce the synergistic effect of the duet which in effect will reduce morbidity and mortality resulting from co-infection.

## Competing interests

The authors wish to state that there is no competing interest whatsoever.

## Authors’ contributions

GP conceived the study, collected samples and drafted the manuscript. SM was involved with sample analyses, data collection, literature search and statistical analysis while SO was involved with sample collection, analyses and review of the manuscript.

## References

[1] Sharma SK, Mohan A, Kadhiravan T. (2005 Apr). HIV-TB co-infection: epidemiology. diagnosis and management. Indian J Med Res.

[2] Egah TE, Okoli CU (2004). Tuberculosis in Jos Nigeria: A 9 year Review of Laboratory Report at Jos University Teaching Hospital (JUTH). Nigeria Med Pract..

[3] De Carvalho BM, Monteiro AJ, Neto RJP, Grangeiro TB, Frota CC (2008). Factors related to HIV/ TB coinfection in a Brazilian Reference Hospital.. BJID..

[4] Onipede AO, Idigbe O, Ako-Nai AK, Omojola O, Oyelese AO, Aboderin AO, Komolafe AO, Wemambu SNC (1999). Seroprevalence of HIV antibodies in TB patients in Ile-Ife.. East Afr Med J..

[5] Iliyasu Z, Babashani M (2009). Prevalence and Predictors of TB coinfection Among HIV seropositive patients attending Aminu Kano Teaching Hospital, Northern Nigeria.. J Epidemiol..

[6] Daniel OJ, Salako AA, Oluwole FA, Alausa OK, Oladapo OT (2004). HIV seroprevalence among newly diagnosed adult PTB patients in Sagamu.. Niger J Med..

[7] Odaibo GN, Gboun MF, Ekanem EE, Gwarzo SN, Saliu I, Egbewunmi SA, Abebe EA, Olaleye DO (2006). HIV infection among patients with PTB in Nigeria.. African Journal of Medicine and Medical Sciences..

[8] Lawson L, Lawson JO, Olajide I, Ememyonu N, Bello CS, Olatunji OO, Davies PD, Thacher TC (2008). Sex differences in the clinical presentation of urban Nigerian patients with pulmonary tuberculosis.. West Afr J Med..

[9] Odaibo GN, Gboun MF, Ekanem EE, Gwarzo SN, Saliu I, Egbewunmi SA, Abebe EA, Olaleye DO (2006). HIV infection among patients with PTB in Nigeria.. African Journal of Medicine and Medical Sciences..

